# A novel mutation in *NLRC4* in a large pedigree with an anakinra responsive autoinflammatory disease

**DOI:** 10.1186/1546-0096-13-S1-P30

**Published:** 2015-09-28

**Authors:** N Volker-Touw, H de Koning, T van Kempen, K Oberndorff, M van Steensel, J Giltay, M Boes, C de Kovel, A Simon, J Frenkel, M van Gijn

**Affiliations:** 1 University Medical Centre Utrecht, Medical Genetics, Utrecht, Netherlands; 2 Radboud University Medical Centre, Department of Dermatology, Nijmegen, Netherlands; 3 University Medical Centre Utrecht, Dept. of Rheumatology and Clinical Immunology, Laboratory of Translational Immunology, Utrecht, Netherlands; 4 Orbis Medical Centre, Department of Pediatrics, Sittard, Netherlands; 5 University of Dundee, Department of Dermatology, Dundee, UK; 6 University Medical Centre Utrecht, Dept of Pediatric Immunology and Laboratory of Translational Immunology, Utrecht, Netherlands; 7 Radboud University Medical Center, Department of General Internal Medicine, Nijmegen, Netherlands; 8 University Medical Centre Utrecht, Department of Pediatrics, Utrecht, Netherlands

## Introduction

Autoinflammatory disorders (AID) are characterized by chronic or recurrent systemic inflammation associated with various clinical presentations. It is a genetically heterogeneous group of diseases. Recently, gain of function mutations in *NLRC4* have been described to be associated with autoinflammatory disease. Here, we report a novel NLRC4 mutation in a large pedigree with an anakinra responsive autoinflammaotry disease.

## Objective

To identify the genetic defect causing an anakinra responsive autoinflammatory syndrome in a large pedigree.

## Patients and methods

We performed whole exome sequencing in the members of a large 6 generation pedigree with an autoinflammatory disease, characterized by recurrent episodes of urticarial skin rash, joint pain and/or swelling, irritation of the eyes, and fatigue. Data with regards to the clinical phenotype were collected retrospectively from the medical charts. Functional studies in monocytes, and histology and immunohistochemical staining in skin biopsies obtained from lesional and uninvolved skin of three patients are currently being performed.

## Results

No mutations were detected in the 20 autoinflammatory associated genes known at the time. Exome sequencing revealed a novel p.Ser445Pro variant in *NLRC4.*The p.Ser445Pro variant segregated with the 13 affected family members. Prediction software programs (Sift, Polyphen) indicate the variant has pathogenic properties. Moreover the variant is located next to the recently described p.His443Pro pathogenic mutation. In all affected family members, the clinical phenotype was influenced by weather conditions, stress, and infection. Severity of the clinical phenotype varied considerably. Moreover, in a subset of the patients, the clinical symptoms resolved promptly after anakinra treatment, indicating involvement of interleukin-1 while other other patients only partially responded to anakinra. Biopsies of lesional skin showed a neutrophilic infiltrate in the dermis. Functional studies in monocytes, and immunohistochemical staining in skin biopsies obtained from lesional and uninvolved skin of patients will provide more insight in the functional effects of the identified *NLRC4* variant.

## Conclusions

In this study we identify and describe a novel variant in *NLRC4* in a large pedigree with an partially anakinra responsive autoinflammatory syndrome, and expanded the clinical phenotype associated with NLRC4-inflammasome mediated autoinflammatory disease.

